# Chronic kidney disease in disadvantaged populations: The case of Africa

**DOI:** 10.4102/phcfm.v7i1.839

**Published:** 2015-04-24

**Authors:** Guillermo Garcia-Garcia, Vivekanand Jha, On behalf of the World Kidney Day Steering Committee

**Affiliations:** 1 Nephrology Service, Hospital Civil de Guadalajara, University of Guadalajara Health Sciences Center, Guadalajara, Jal. Mexico; 2 Postgraduate Institute of Medical Education and Research, Chandigarh, India; 3 George Institute for Global Health, New Delhi, India and University of Oxford, United Kingdom

‘Of all of the forms of inequality, injustice in health is the most shocking and inhumane.’

(Dr. Martin Luther King, Jr)

Disadvantaged communities have a disproportionate burden of unrecognised and untreated chronic kidney disease (CKD).^1^ A position of disadvantage negatively influences healthy behaviours, health care access and environmental exposure, but reduces access to goods and services, in particular clean water and sanitation, information about preventive behaviours and adequate nutrition.^2^

Africa, especially sub-Saharan Africa, remains the most disadvantaged in terms of access to nephrology services for the population. Except for two countries – Nigeria and South Africa – all others have less than 10 nephrologists.

Chronic glomerulonephritis and interstitial nephritis remain amongst the principal causes of CKD, reflecting the high prevalence of infections that affect the kidneys. Several parasitic infections cause CKD through ureteric obstruction (schistosomiasis), interstitial nephritis (kala-azar) and glomerulonephritis (malaria).^3,4,5^ However, diabetes mellitus (DM) and hypertension are emerging as the leading causes of CKD in many African countries. The estimated increase in DM2 in Africa is anticipated to be 12.7 million, an increase of 140%, by 2025. It is expected that the prevalence of diabetic nephropathy, currently estimated to be 6% – 16%, will increase along with this estimation.^5,6^

In addition, in sub-Saharan Africa, HIV-associated nephropathy has emerged as a public health problem in the last two decades. The reported prevalence of CKD in HIV-infected antiretroviral treatment (ART)-naïve patients in the region ranges from 6 to 45%. An escalating burden of HIV CKD may be anticipated in the near future, because of the increasing life expectancy of patients on ART, aging of HIV-infected populations and nephrotoxicity of the various drugs used in this population.^5^ In addition, North African countries have reported a high prevalence of CKD of unknown aetiology. The cause seems to be associated with environmental pollution.^6^

Access to renal replacement therapy (RRT) is dependent mostly on healthcare expenditures and economic strength. RRT prevalence and kidney transplantation rates correlate significantly with gross national income and health expenditure. Haemodialysis is the treatment modality. Less than 5% of all dialysis patients are on peritoneal dialysis (PD). Amongst the factors identified against PD are the elevated costs of dialysis fluids and a perception of a high risk of peritonitis. Transplantation activity falls short of demand, with inadequate financial support and lack of an organised deceased donor transplant programme being the major obstacles. Except for South Africa, the vast majority of grafts come from living donors. Deceased donors are poorly utilised because of an ineffective organ procurement network, lack of facilities for taking care of potential donors and poor public education.^4,5,7^

Overall treatment outcomes are relatively poor amongst Africans who receive RRT and many patients are underdialysed. Since the majority of cases are self-funded, very few are able to sustain dialyisis beyond six months and have to stop dialysis when funds are depleted.^5^ Although graft and patient survival are not generally available for the region, a recent report from Nigeria found that graft and patient survival were similar to those of African Americans in the United States.^7^

Human resources for renal care remain at a critical level in many parts of Africa. Nephrologist rates per million population (pmp) range from 0.5 pmp in Kenya to 1.1 pmp in South Africa. In many countries, there are fewer than 10 nephrologists. Similarly, the number of nurses and dialysis technicians are also inadequate. The shortage is aggravated by the continuous ‘brain drain’ of health workers from Africa to more affluent nations.^5,8^

The aforementioned places a special burden on the professional community and policymakers to ensure provision of appropriate renal care to the African population. This requires actions such as expanding the reach of dialysis through development of low-cost alternatives that can be practised in remote locations and by implementation and evaluation of cost-effective prevention strategies. Kidney transplantation should be promoted by expanding deceased donor transplant programmes and use of inexpensive, generic immunosuppressive drugs. The International Society of Nephrology is addressing these challenges through its Research and Prevention Committee as well as its Dialysis and Educational programmes.

The message of World Kidney Disease 2015 is that a concerted attack against the diseases that lead to end-stage kidney disease, by increasing community outreach, better education, improved economic opportunity and access to preventive medicine for those at highest risk, could end the unacceptable relationship between CKD and disadvantage in these communities.
